# Estimation of Improvements in Mortality in Spectrum Among Adults With HIV Receiving Antiretroviral Therapy in High-Income Countries

**DOI:** 10.1097/QAI.0000000000003326

**Published:** 2024-01-04

**Authors:** Adam Trickey, Robert Glaubius, Nikos Pantazis, Robert Zangerle, Linda Wittkop, Janne Vehreschild, Sophie Grabar, Matthias Cavassini, Ramon Teira, Antonella d’Arminio Monforte, Jordi Casabona, Ard van Sighem, Inma Jarrin, Suzanne M. Ingle, Jonathan A. C. Sterne, Jeffrey W. Imai-Eaton, Leigh F. Johnson

**Affiliations:** a Population Health Sciences, University of Bristol, Bristol, United Kingdom;; b Center for Modeling, Planning and Policy Analysis, Avenir Health, Glastonbury, CT;; c Department of Hygiene, Epidemiology and Medical Statistics, Medical School, National and Kapodistrian University of Athens, Athens, Greece;; d Department of Dermatology, Venereology and Allergy, Medical University Innsbruck, Innsbruck, Austria;; e Univ. Bordeaux, INSERM, Institut Bergonié, BPH, U1219, CIC-EC 1401, Bordeaux, France;; f INRIA SISTM Team, Talence, France;; g CHU de Bordeaux, Service d'information médicale, INSERM, Institut Bergonié, CIC-EC 1401, Bordeaux, France;; h Department I for Internal Medicine, University Hospital of Cologne, Cologne, Germany;; i Sorbonne Université, INSERM, Institut Pierre Louis d’Épidémiologie et de Santé Publique (IPLESP), Paris, France;; j Department of Public Health, AP-HP, St Antoine Hospital, Paris, France;; k Infectious Diseases Service, Lausanne University Hospital and University of Lausanne, Lausanne, Switzerland;; l Servicio de Medicina Interna, Hospital Universitario de Sierrallana, Torrelavega, Cantabria, Spain;; m Department of Health Sciences, Clinic of Infectious and Tropical Diseases, University of Milan, Milan, Italy;; n Centre d'Estudis Epidemiològics sobre la SIDA i les ITS de Catalunya (CEEISCAT), Institut de Recerca en Ciències de la Salut Germans Trias i Pujol (IGTP), Campus de Can Ruti, Badalona, Catalonia, Spain;; o Stichting HIV Monitoring, Amsterdam, The Netherlands;; p Centro Nacional de Epidemiología, Instituto de Salud Carlos III, Madrid, Spain;; q CIBER de Enfermedades Infecciosas, Instituto de Salud Carlos III, Madrid, Spain;; r National Institute for Health and Care Research Bristol Biomedical Research Centre, Bristol, United Kingdom;; s Health Data Research UK South-West, Bristol, United Kingdom;; t MRC Centre for Global Infectious Disease Analysis, School of Public Health, Imperial College London, London, United Kingdom;; u Center for Communicable Disease Dynamics, Department of Epidemiology, Harvard T.H. Chan School of Public Health, Boston, MA; and; v Centre for Infectious Disease Epidemiology and Research, School of Public Health, University of Cape Town, Cape Town, South Africa.

**Keywords:** AIDS mortality, excess mortality, modelling, parameters, Europe, high income

## Abstract

**Introduction:**

Mortality rates for people living with HIV (PLHIV) on antiretroviral therapy (ART) in high-income countries continue to decline. We compared mortality rates among PLHIV on ART in Europe for 2016–2020 with Spectrum's estimates.

**Methods:**

The AIDS Impact Module in Spectrum is a compartmental HIV epidemic model coupled with a demographic population projection model. We used national Spectrum projections developed for the 2022 HIV estimates round to calculate mortality rates among PLHIV on ART, adjusting to the age/country distribution of PLHIV starting ART from 1996 to 2020 in the Antiretroviral Therapy Cohort Collaboration (ART-CC)'s European cohorts.

**Results:**

In the ART-CC, 11,504 of 162,835 PLHIV died. Between 1996–1999 and 2016–2020, AIDS-related mortality in the ART-CC decreased from 8.8 (95% CI: 7.6 to 10.1) to 1.0 (0.9–1.2) and from 5.9 (4.4–8.1) to 1.1 (0.9–1.4) deaths per 1000 person-years among men and women, respectively. Non–AIDS-related mortality decreased from 9.1 (7.9–10.5) to 6.1 (5.8–6.5) and from 7.0 (5.2–9.3) to 4.8 (4.3–5.2) deaths per 1000 person-years among men and women, respectively. Adjusted all-cause mortality rates in Spectrum among men were near ART-CC estimates for 2016–2020 (Spectrum: 7.02–7.47 deaths per 1000 person-years) but approximately 20% lower in women (Spectrum: 4.66–4.70). Adjusted excess mortality rates in Spectrum were 2.5-fold higher in women and 3.1–3.4-fold higher in men in comparison to the ART-CC's AIDS-specific mortality rates.

**Discussion:**

Spectrum's all-cause mortality estimates among PLHIV are consistent with age/country-controlled mortality observed in ART-CC, with some underestimation of mortality among women. Comparing results suggest that 60%–70% of excess deaths among PLHIV on ART in Spectrum are from non-AIDS causes.

## Abstract 2

Supplemental Digital Content is Available in the Text.

## INTRODUCTION

Before combination antiretroviral therapy (ART) was introduced across multiple countries in 1996, persons living with HIV (PLHIV) in Western and Central Europe and North America (WCENA) had very high rates of mortality, mostly because of AIDS.^1–3^ Mortality rates have subsequently decreased as PLHIV have started ART and successfully suppressed viral replication, which has reduced the risk of AIDS and death.^4^ Decreasing on-ART mortality rates among PLHIV in WCENA has meant that PLHIV in those settings are aging and there is less AIDS-related mortality, whereas mortality because of other causes that are increasingly common in the aging general population (eg, cardiovascular disease, cancer) is increasing.^5^ Among PLHIV in WCENA, substance use and comorbidities such as hepatitis C virus are more common than in the general population, so there are increased mortality rates because of such non–AIDS-related causes.^6^

Most countries prepare annual estimates of key HIV indicators, including HIV-related deaths, using the Spectrum model.^7^ Spectrum estimates are used for national and global planning and are published annually by the UNAIDS.^8^ Estimated numbers of deaths from any cause among PLHIV on ART depend on routinely collected program data on national ART patient cohort sizes, prevailing rates of mortality from HIV-unrelated causes,^9^ and input rates of excess mortality among PLHIV on ART. Recommended default excess mortality rates were estimated from treatment cohort studies,^10,11^ which are commissioned by the UNAIDS for use in Spectrum. Spectrum estimates of excess deaths among PLHIV on or off ART are often reported as HIV-related or AIDS-related deaths,^12^ although, many of these excess deaths are not HIV related.

As mortality rates for PLHIV on ART in high-income countries continue to drop, Spectrum may overestimate on-ART mortality because of using older parameter values, particularly in the WCENA region. Several countries in this region have used lower, non-default HIV-related mortality rates on ART within their Spectrum files to compensate for this overestimation of mortality, which prompted the present review to determine whether these default rates should be revised.

We aimed to review AIDS-related and non–AIDS-related mortality rates over time using data from a collaboration of cohorts of PLHIV on ART in Europe and compare the estimated mortality rates for 2016–2020 with the estimates produced by Spectrum.

## METHODS

### Antiretroviral Therapy Cohort Collaboration Data

Data were taken from the Antiretroviral Therapy Cohort Collaboration (ART-CC), a collaboration of HIV cohort studies from Europe and North America.^13^ Ethics committees or institutional review boards approved the individual cohorts, which used standardized data collection methods and regularly followed up patients. Cohorts gathered information on mortality through linkage with vital statistics agencies and hospitals or physician report, and the active follow-up of participants. For these analyses, European ART-CC cohorts were included if they had assigned a cause to ≥70% of deaths among PLHIV who were eligible to be included in the ART-CC baseline data set; 11 of 13 of the ART-CC's European cohorts were included. The included cohorts are listed in Appendix Table 1, Supplemental Digital Content, http://links.lww.com/QAI/C187. Only data from the ART-CC's European cohorts were used and not from the ART-CC's North American cohorts because the North American AIDS Cohort Collaboration on Research and Design was deemed to hold more representative data for PLHIV in North America.^14^ The eligibility criteria for PLHIV to be included in the ART-CC baseline data set were having started combination ART (3 drugs or more) with no prior exposure to ART medications when aged ≥16 years old and with a CD4 cell count and HIV-1 RNA viral load measurement taken within a window of 3 months before to 1 week after starting combination ART.

We adapted the Cause of Death (CoDe) project protocol^15^ to classify causes of death information into a single cause in the HICDEP format (https://hicdep.org/Wiki/v/10/pt/3/Table/104/FieldID/1321). Information on cause of death was recorded either as International Classification of Diseases, Ninth Revision (ICD-9) or 10th Revision (ICD-10), or free text. If ICD-9 or ICD-10 codes were available, causes of death were classified by a clinician and a computer algorithm.^5^ When ICD-9 or ICD-10 codes were not available, 2 clinicians independently classified each death. Disagreements between clinicians and/or computer-assigned codes were resolved through panel discussion. Further information on this process has been detailed elsewhere.^16^ For comparison with Spectrum, causes of death were categorized as AIDS related or non–AIDS related. Deaths were coded as AIDS related if there was a serious AIDS defining condition before death and/or a low CD4 count (<100/μL) within a year (18 months if off treatment) of death, and a diagnosis compatible with AIDS as cause of death.^5^ All other deaths, including those of unknown cause, were considered non–AIDS related. The cause of death coding rules is included in the Appendix, Supplemental Digital Content, http://links.lww.com/QAI/C187.

### The Spectrum Model

The AIDS Impact Module in Spectrum is a compartmental HIV epidemic model that is coupled with a demographic population projection method.^7,17^ For the 2022 HIV estimates round, most countries used demographic inputs to Spectrum, including country-specific life tables, that were derived from the 2019 World Population Prospects.^9^ PLHIV aged 15 years and older are stratified by sex, age, CD4 cell count category (CD4>500, 350–500, 250–349, 200–249, 100–199, 50–99, <50 cells/mm^3^), and ART status. Excess mortality rates among PLHIV on ART vary by sex, current age (15–24, 25–34, 35–44, ≥45), CD4 category at ART initiation, and time since ART initiation (<6, 6–11, or ≥12 months). Recommended default excess mortality rate inputs among PLHIV on ART were estimated using data on all-cause mortality^10,11^ and subtracting background mortality (population-level average mortality from HIV-unrelated causes) by age, sex, and calendar year.

Spectrum model assumptions are routinely reviewed by the UNAIDS Reference Group on Estimates, Modeling, and Projections.^18^ Appendix Table 4, Supplemental Digital Content, http://links.lww.com/QAI/C187 and the subsequent sections in the appendix review the mortality rates used for PLHIV starting ART with CD4 counts >500 cells/mm^3^.

### Statistical Analyses

ART-CC cohort data were split into calendar periods of follow-up (1996–1999, 2000–2003, 2004–2007, 2008–2011, 2012–2015, and 2016–2020; with follow-up in 2020 ending in April 2020 before the impact of the COVID-19 epidemic), for which we calculated time-updated characteristics for the beginning of each period if the person did not start ART during that period. The characteristics were used to describe the ART-CC population. For comparability with Spectrum, we removed follow-up of PLHIV when they were aged <20 years. We calculated crude rates of AIDS-related and non–AIDS-related mortality per follow-up period, stratified by sex and by time-updated age category. Cohort data analyses were performed in Stata v17.1 (StataCorp, 2021) (Table 1).

**TABLE 1.** Time-Updated Characteristics of PLHIV on ART in ART-CC Analyses

Characteristics	Follow-up Years					
	1996–1999	2000–2003	2004–2007	2008–2011	2012–2015	2016–2020
N	19,271	40,496	63,484	93,394	119,912	126,857
Median age (IQR), yr	35 (31–42)	36 (31–43)	39 (33–45)	41 (34–47)	42 (35–50)	45 (36–52)
Sex/acquisition group, n (%)						
MSM	6612 (34.3)	12,684 (31.3)	20,845 (32.8)	36,520 (39.1)	53,280 (44.4)	58,784 (46.3)
Male; IDU	3232 (16.8)	5541 (13.7)	6832 (10.8)	7490 (8.0)	7530 (6.3)	6638 (5.2)
Female; IDU	919 (4.8)	1589 (3.9)	1949 (3.1)	2119 (2.3)	2118 (1.8)	1838 (1.5)
Male; heterosexual	3569 (18.5)	8371 (20.7)	13,324 (21.0)	18,985 (20.3)	23,503 (19.6)	24,461 (19.3)
Female; heterosexual	3430 (17.8)	9083 (22.4)	15,867 (25.0)	22,354 (23.9)	26,448 (22.1)	27,750 (21.9)
Either sex; other/unknown	1509 (7.8)	3228 (8.0)	4667 (7.4)	5926 (6.4)	7033 (5.9)	7386 (5.8)
ART naive, n (%)*	19,271 (100.0)	22,573 (55.7)	26,870 (42.3)	35,916 (38.5)	36,648 (30.6)	20,320 (16.0)
CD4 cell count, cells (%)						
0–199	8205 (42.6)	14,978 (37.0)	17,179 (27.1)	16,061 (17.2)	13,850 (11.6)	9390 (7.4)
200–349	4827 (25.1)	10,294 (25.4)	17,655 (27.8)	23,880 (25.6)	19,083 (15.9)	13,335 (10.5)
350–499	3484 (18.1)	6005 (14.8)	10,286 (16.2)	20,533 (22.0)	26,680 (22.3)	20,724 (16.3)
≥500	2755 (14.3)	8068 (19.9)	14,784 (23.3)	27,127 (29.1)	52,747 (44.0)	73,976 (58.3)
Missing	0 (0.0)	1151 (2.8)	3580 (5.6)	5793 (6.2)	7552 (6.3)	9432 (7.4)
Country, n (%)						
Austria	413 (2.1)	997 (2.5)	1703 (2.7)	2639 (2.8)	3518 (2.9)	3664 (2.9)
France	10,401 (53.6)	21,509 (52.9)	32,676 (51.3)	45,707 (48.8)	56,013 (46.6)	59,668 (46.9)
Germany	173 (0.9)	435 (1.1)	779 (1.2)	1252 (1.3)	1524 (1.3)	1423 (1.1)
Greece	288 (1.5)	682 (1.7)	1266 (2.0)	2515 (2.7)	3738 (3.1)	3342 (2.6)
Italy	1585 (8.2)	2386 (5.9)	2537 (4.0)	3451 (3.7)	6421 (5.3)	8421 (6.6)
Netherlands	1656 (8.5)	3876 (9.5)	6634 (10.4)	10,180 (10.9)	13,680 (11.4)	14,656 (11.5)
Spain	3134 (16.2)	7673 (18.9)	13,791 (21.7)	22,014 (23.5)	28,527 (23.7)	29,272 (23.0)
Switzerland	1621 (8.4)	2938 (7.2)	4098 (6.4)	5636 (6.0)	6491 (5.4)	6411 (5.0)

IDU, injecting drug use; IQR, interquartile range; MSM, men who have sex with men.

* ART naive when first contributing follow-up to the period.

We calculated equivalent population mortality rates stratified by sex and age (in 5-year categories) for each follow-up period to correspond with the country mix in the ART-CC data. Mortality rates for each age and sex group were retrieved for each country included in the analyses using data from mortality.org for 2014.^19^ The retrieved country-specific mortality rates for each age/sex group were then multiplied by the proportion of PLHIV in each age/sex group from the country that made up the entire ART-CC sample for each follow-up period.

### Spectrum Mortality Rates on ART

We used national Spectrum projections to calculate all-cause mortality rates among PLHIV on ART and in the general population for comparison to ART-CC estimates. We used national projections developed for the 2022 HIV estimates round for the 8 countries with ART-CC cohorts. Five projections (France, Greece, Italy, Netherlands, Switzerland) were shared by national HIV estimate teams and are available from the UNAIDS on request.^20^ Three other projections (Austria, Germany, Spain) contributed to regional and global UNAIDS analyses but are not publicly available. Five of these projections (France, Greece, Italy, Netherlands, and Spain) used nondefault mortality rate inputs that were lower than regional Spectrum defaults. To assess the adequacy of regional defaults, projections were modified for this analysis to use Spectrum's regional default on-ART mortality rates. Rates were calculated using Spectrum estimates for 2016–2019 because ART-CC follow-up during 2020 was minimal.

We report all-cause mortality rates $m^{A}\left(t;s\right)$ for people on ART aged 20 years and above:

$$m^{A}\left(t;s\right)=\frac{\sum_{k}\sum_{x=20}^{80+}\left[\frac{D^{A}\left(t;k,s,x\right)}{N^{A}\left(t;k,s,x\right)}+\frac{D^{B}\left(t;k,s,x\right)}{N\left(t;k,s,x\right)}\right]W^{A}\left(t;k,s,x\right)}{\sum_{k}\sum_{x=20}^{80+}W^{A}\left(t;k,s,x\right)}.$$

Spectrum estimates of excess deaths on ART ($D^{A}$), HIV-unrelated deaths in the overall population regardless of HIV status ($D^{B}$), and population sizes on ART ($N^{A}$) or overall (N) were stratified by year t, country k, sex s, and age x. The ratio $D^{A}/N^{A}$ reflects excess mortality among PLHIV on ART, whereas $D^{B}/N\;$ reflects HIV-unrelated mortality. To account for differences in demographic characteristics between Spectrum and the ART-CC, we evaluated 3 sets of weights $W^{A}$: Spectrum's projected population on ART (1) as-is or adjusted to match the observed ART-CC distribution of patients (2) by age, or (3) by age and country.

We calculated equivalent general population all-cause mortality rates by sex m(t;s) analogously:

$$m\left(t;s\right)=\frac{\sum_{k}\sum_{x=20}^{80+}\frac{D^{A}\left(t;k,s,x\right)+D^{H}\left(t;k,s,x\right)+D^{B}\left(t;k,s,x\right)}{N\left(t;k,s,x\right)}W^{A}\left(t;k,s,x\right)}{\sum_{k}\sum_{x=20}^{80+}W^{A}\left(t;k,s,x\right)}.$$

Here, $D^{H}\left(t;k,s,x\right)$ are HIV-related deaths among PLHIV not on ART.

We calculated model all-cause mortality rates in R v4.2.2 using estimated population sizes and numbers of deaths extracted from Spectrum files submitted for the 2022 HIV estimates round.

### Sensitivity Analysis

To investigate the effect of removing the ART-CC inclusion criteria on the age of the cohort, we calculated the median age across all PLHIV alive as of January 1, 2016, that the ART-CC holds data on. PLHIV from the same cohorts included in the main analysis were again included but CD4 count and viral load values around ART start, prior ART status, and loss-to-follow-up before January 1, 2016, were disregarded.

## RESULTS

### Characteristics of ART-CC Population

Overall, follow-up from 162,835 PLHIV was included in the analysis, with the number of PLHIV in follow-up increasing from 19,271 in 1996–1999 to 126,857 in 2016–2020. The median age of PLHIV at the start of the follow-up period was 35 years (interquartile range: 31–42 years) in 1996–1999, increasing to 45 years (36–52 years) in 2016–2020. There was a smaller increase in age when starting ART from 35 years (31–42 years) in 1996–1999 to 38 years (30–47 years) in 2016–2020. In a sensitivity analysis removing the ART-CC eligibility criteria, the median age of the cohort increased to 48 years (39–55 years) in 2016–2020. The percentage of PLHIV in follow-up who acquired HIV through injecting drug use decreased over time, whereas the percentage of men who acquired HIV through sex with men increased. The percentage of PLHIV who started each follow-up period as ART naive reduced over time from 100.0% in 1996–1999 to 16.0% in 2016–2020, whereas the percentage of PLHIV who started each follow-up period with CD4 counts of 0–199 decreased from 42.6% in 1996–1999 to 7.4% in 2016–2020.

**FIGURE 1.**
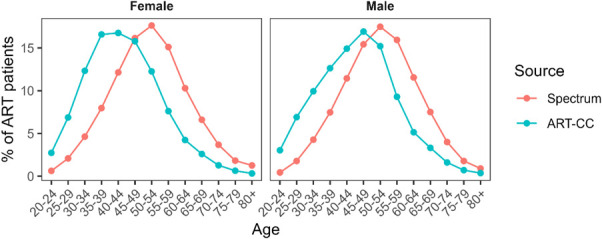
Age distribution of PLHIV on ART in the ART-CC and Spectrum, 2016–2019.

Spectrum projected a cumulative 1.95 million person-years on ART during 2016–2019. Men accounted for 74% of person-years lived by PLHIV. PLHIV on ART were older in the Spectrum projections than in the ART-CC (Fig. 1). The median age of PLHIV on ART was 53 years for men and 52 years for women, about 7 years older than the ART-CC median for 2016–2020.

### AIDS and Non-AIDS Mortality in the ART-CC

There were 11,504 deaths in 1,100,155 person-years of follow-up, of which 2648 deaths (23.0%) were classified as AIDS related and 8856 (77.0%) were classified as non–AIDS related. In 1996–1999, 226 of 466 deaths (48.5%) were classified as AIDS related, compared with 359 of 2348 deaths (15.3%) in 2016–2020.

Table 2 shows cause-specific mortality rates over time for men and women, whereas Appendix Tables 2 and 3, Supplemental Digital Content, http://links.lww.com/QAI/C187 show the age-stratified cause-specific mortality rates over 2016–2020 for men and women on ART, respectively. Both AIDS-related and non–AIDS-related mortality rates declined between 1996–1999 and 2016–2020 for men and women on ART. The largest decreases were seen for AIDS-related mortality among men, from 8.8 (95% confidence interval: 7.6–10.1) to 1.0 (0.9–1.2) deaths per 1000 person-years, but AIDS-related mortality also decreased substantially for women, from 5.9 (4.4–8.1) to 1.1 (0.9–1.4) deaths per 1000 person-years. During 1996–1999, approximately half of mortality among men and women on ART was classified as AIDS related, with rates of non-AIDS mortality per 1000 person-years of 9.1 (7.9–10.5) and 7.0 (5.2–9.3) among men and women, respectively. A far lower percentage of mortality was classified as AIDS related in 2016–2020, with non-AIDS mortality rates having fallen more slowly than AIDS-related mortality to 6.1 (5.8–6.5) and 4.8 (4.3–5.2) deaths per 1000 person-years among men and women on ART, respectively.

**TABLE 2.** AIDS and Non-AIDS Mortality Rates per 1000 Person-Years in the ART-CC Over Time, Stratified by Sex

Periods	AIDS Mortality		Non-AIDS Mortality		General Population	
	Men	Women	Men	Women	Men	Women
1996–1999	8.8 (7.6–10.1)	5.9 (4.4–8.1)	9.1 (7.9–10.5)	7.0 (5.2–9.3)	1.8	0.8
2000–2003	5.1 (4.6–5.6)	3.6 (3.0–4.4)	9.8 (9.2–10.5)	6.2 (5.3–7.1)	2.1	0.8
2004–2007	3.0 (2.7–3.3)	1.8 (1.5–2.2)	8.9 (8.4–9.4)	5.0 (4.4–5.6)	2.5	0.9
2008–2011	2.1 (1.9–2.4)	1.3 (1.1–1.6)	7.6 (7.2–8.0)	4.3 (3.9–4.8)	2.9	1.1
2012–2015	1.4 (1.3–1.6)	1.2 (1.0–1.5)	6.8 (6.5–7.1)	4.7 (4.3–5.1)	3.3	1.5
2016–2020	1.0 (0.9–1.2)	1.1 (0.9–1.4)	6.1 (5.8–6.5)	4.8 (4.3–5.2)	4.1	1.9

As the ART-CC population aged during this time, mortality rates in equivalent aged-matched general population cohorts of men and women increased between 1996–1999 and 2016–2020, from 1.8 to 4.1 and from 0.8 to 1.9 deaths per 1000 person-years in men and women, respectively.

### Comparison of Spectrum to the ART-CC

**FIGURE 2.**
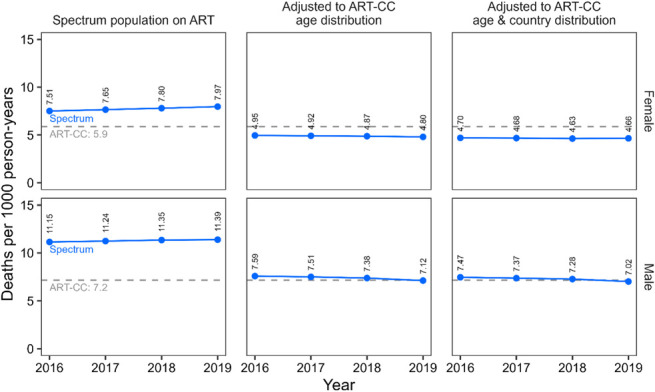
Comparing all-cause mortality rates per 1000 person-years for PLHIV on ART calculated for the ART-CC and Spectrum, 2016–2019. Spectrum mortality rates are calculated for either the modeled ART population or adjusted to match ART-CC characteristics by age alone or age and country simultaneously. Spectrum mortality rates are calculated for each year, whereas ART-CC mortality rates are calculated for the overall period.

Spectrum estimated that 8200 excess deaths occurred among PLHIV on ART during 2016–2019. All-cause mortality rates on ART increased from 11.15 to 11.39 deaths per 1000 person-years in men and from 7.51 to 7.97 deaths per 1000 person-years in women during 2016–2019 (Fig. 2), higher than contemporary rates in ART-CC by 28%–36% in women and by 56%–59% in men. Compared with unadjusted rates, adjustment to the ART-CC population by age reduced all-cause mortality rates on ART by 32%–40% and by 33%–42% when adjusted by both age and country. After these adjustments, all-cause mortality rates in Spectrum among men were close to ART-CC estimates for 2016–2020 (Spectrum: 7.02–7.47 deaths per 1000 person-years; ART-CC 7.2) but 20% lower in women (Spectrum: 4.66–4.70; ART-CC 5.9 deaths per 1000 person-years).

**FIGURE 3.**
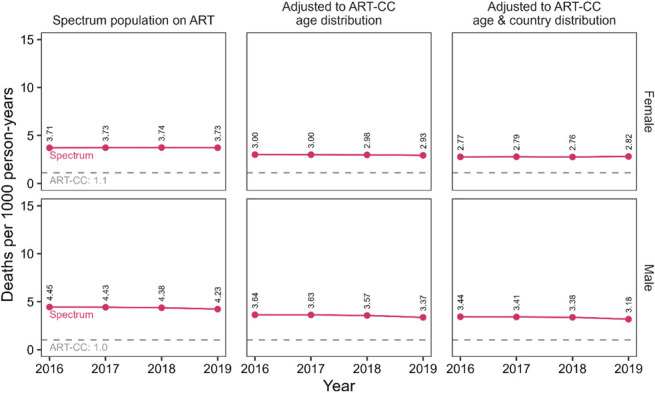
Comparing excess mortality rates per 1000 person-years for PLHIV on ART in Spectrum to AIDS-specific mortality rates for the ART-CC, 2016–2019. Spectrum mortality rates are calculated for either the modeled ART population or adjusted to match ART-CC characteristics by age alone or age and country simultaneously.

Rates of excess mortality (ie, with HIV-unrelated mortality excluded) among PLHIV on ART in Spectrum ranged from 4.23 to 4.45 and 3.71–3.74 deaths per 1000 person-years in men and women, respectively (Fig. 3). Excess mortality rates decreased by 23%–26% after adjustment to the ART-CC population by age and country. After this adjustment, excess mortality rates in Spectrum were 2.5-fold in women and 3.1- to 3.4-fold higher in men in comparison to AIDS-specific mortality rates in the ART-CC (women, 1.1; men, 1.0 deaths per 1000 person-years).

Differing assumptions about mortality in the general population may contribute to differences in all-cause mortality rates on ART between Spectrum and the ART-CC. General population all-cause mortality rates in Spectrum were higher than general population estimates corresponding to the demographics of the ART-CC by 99%–122% in men and 64%–75% in women (see Figure 1, Supplemental Digital Content, http://links.lww.com/QAI/C187). These general population mortality rates were similar after adjusting Spectrum estimates to ART-CC age and country characteristics.

## DISCUSSION

Mortality on ART in WCENA has decreased between 1996 and 2020 and non-AIDS mortality now makes up most deaths for PLHIV on ART. All-cause mortality rates among PLHIV on ART estimated by Spectrum for 2016–2019 are 28%–59% higher than contemporary ART-CC estimates. This raised concerns that Spectrum estimates may not reflect the decreasing mortality among PLHIV observed in the ART-CC, which was addressed in some national HIV estimates by reducing age-specific mortality rate inputs to Spectrum. However, most of the difference in overall all-cause mortality between ART-CC and Spectrum is because of the age distributions of PLHIV on ART, not differences in age-specific mortality. The median age of ART patients in Spectrum was approximately 7 years older than in those in ART-CC during 2016–2019. National HIV estimates teams are encouraged to validate the modeled age distribution of PLHIV on ART against routine program data when preparing their HIV estimates and to adjust model inputs to reduce discrepancies. Nevertheless, we were not able to determine whether the modeled age distribution in Spectrum was representative of national treatment cohorts because these data were not available to us. This knowledge gap limits our ability to determine what changes to Spectrum might be needed given the observed differences in age difference between Spectrum and ART-CC. Age- and country-matched excess mortality rates in Spectrum were 2.5-fold in women and 3.1–3.4-fold higher in men in comparison to AIDS-specific mortality rates in the ART-CC. This suggests that a considerable proportion of the excess mortality in Spectrum is because of non-AIDS causes. For the 2023 HIV estimates round, we updated Spectrum to allow direct comparison of estimated all-cause deaths on ART to national vital registration data, and regional HIV estimates workshops emphasized performing validation checks. In addition, in the latest version of Spectrum, the mortality assumptions for PLHIV starting ART with CD4 count of >500 cells per cubic millimeter will be same as for PLHIV starting with CD4 count of 350–499 cells per cubic millimeter.

### Comparison With Other Literature

That mortality because of non–AIDS-related causes among PLHIV on ART in high-income countries now forms the majority of deaths has been detailed elsewhere. A systematic review and meta-analysis of studies between 2005 and 2016 by Farahani et al^21^ found that in high-income countries, 54% (95% CI: 46% to 62%) of mortality among PLHIV on ART was because of non-AIDS causes. A Spanish population-based cohort of PLHIV, including those not on ART, found that all-cause mortality decreased from 1999 to 2018, with the decrease driven by AIDS-related mortality, whereas non-AIDS mortality remained stable and, therefore, accounted for a higher proportion of mortality.^22^ A study among PLHIV on ART in British Columbia, Canada, observed reductions in all-cause, AIDS-related, and certain types of non–AIDS-related mortality over calendar time from 2001 to 2012, with non–AIDS-related mortality accounting for most deaths by 2011–2012.^23^ Reductions in AIDS-related mortality are often attributed to the increased effectiveness and availability of ART; however, deaths described as non–AIDS-related also declined when ART became available.^1^ PLHIV on ART continue to have a higher prevalence of many non–AIDS-related comorbidities than the general population.^24^ This is partly because of lifestyle factors, such as a higher prevalence of smoking among PLHIV.^25^ However, the ART-CC has previously detailed that increased non-AIDS mortality is seen among PLHIV on ART that have AIDS-defining events.^26^ In addition, markers of inflammation that are associated with increased non–AIDS-related mortality are often elevated among PLHIV, even when on ART,^27,28^ which indicates that HIV also leads through multiple mechanisms to an increase in what is described as non-AIDS mortality.

### Strengths and Limitations

A strength of these analyses was the use of a large, multicountry data set of PLHIV on ART that should be quite generalizable to the populations of PLHIV on ART in Western European countries. Previous research comparing European HIV cohort data with surveillance system data found that although the cohorts were generally representative of the HIV-diagnosed populations in the surveillance system, some important subgroups, such as PLHIV aged ≥55 years, were underrepresented.^29^ Although the population included should be quite generalizable to these countries, the ART-CC inclusion criteria (PLHIV starting combination ART as ART naive with CD4 counts and viral load measurements within a month of ART initiation^13^) may mean that the population used here is slightly younger than the overall populations on ART in these countries. We investigated this in a sensitivity analysis and found that when removing these inclusion criteria, a 4-year difference remained in comparison to Spectrum. However, this could have been affected by the individual cohorts selecting records of PLHIV to be sent to the ART-CC that they believed matched the ART-CC's eligibility criteria, that is, they were not ART naive when starting combination ART. Another strength of these analyses is the use of detailed data on causes of death. However, ascertaining a single cause of death can be complicated and was not possible for approximately 20% of the deaths, particularly in cases where there were multiple major comorbidities, but these cases should mostly be where there are competing non-AIDS conditions, so should not affect the interpretation of this analysis. Interpreting mortality rates for PLHIV in comparison with an age-, sex-, and country-matched general population can be difficult because of differences in other confounders, such as the prevalence of comorbidities and substance use issues. These country-matched general population mortality rates were taken for 2014 rather than using separate rates for each calendar year period. However, although mortality rates in WCENA countries between 1996 and 2020 will have decreased over this period, the magnitude will have been small compared with the changes in mortality rates among PLHIV.

### Implications for National HIV Estimates Using Spectrum

Reported estimates of AIDS-related deaths prepared using Spectrum should be clarified. HIV-related mortality rate inputs to Spectrum reflect excess mortality among people living with HIV, but the corresponding deaths estimates are usually reported as AIDS-related deaths, which might be interpreted as deaths caused by AIDS.^12^ Differing age distributions between the ART-CC and Spectrum inputs caused differences in the overall mortality rates. However, we found that Spectrum produces estimates of all-cause mortality among PLHIV that are consistent with mortality observed in ART-CC after controlling for age and country. We found that a substantial proportion of excess mortality among PLHIV on ART in Europe is from non-AIDS causes and that excess mortality rates among PLHIV on ART in Spectrum are 2.5–3.4-fold higher than AIDS-related deaths rates in the ART-CC. This suggests that 60%–70% of excess deaths among PLHIV on ART in Spectrum are from non-AIDS causes. Thus, misinterpreting excess deaths as AIDS-related deaths risks overstating the numbers of deaths that might be prevented through ART, although ART should also reduce some deaths that are currently classified as non-AIDS mortality. Misinterpreting excess deaths as AIDS-related could also underemphasize other interventions targeted at reducing the apparent burden of non-AIDS mortality associated with HIV. Second, different age distributions of the PLHIV on ART was a major reason for differences in crude mortality among PLHIV between ART-CC and modeled Spectrum estimates. Estimate teams should prioritize review demographic characteristics of modeled HIV populations to ensure accurate HIV estimates.

## Supplementary Material

SUPPLEMENTARY MATERIAL

## References

[1] Vyas KJ Marconi VC Moanna A . Trends in cause-specific mortality among Veterans with HIV: a 35-year (1982-2016) analysis of the HIV Atlanta VA cohort study. J Acquir Immune Defic Syndr. 2023;92:17–26. 36166297 10.1097/QAI.0000000000003107 PMC9742180

[2] Krentz HB Kliewer G Gill MJ. Changing mortality rates and causes of death for HIV-infected individuals living in Southern Alberta, Canada from 1984 to 2003. HIV Med. 2005;6:99–106. 15807715 10.1111/j.1468-1293.2005.00271.x

[3] Mocroft A Vella S Benfield TL . Changing patterns of mortality across Europe in patients infected with HIV-1. EuroSIDA Study Group. Lancet. 1998;352:1725–1730. 9848347 10.1016/s0140-6736(98)03201-2

[4] Antiretroviral Therapy Cohort Collaboration. Survival of HIV-positive patients starting antiretroviral therapy between 1996 and 2013: a collaborative analysis of cohort studies. Lancet HIV. 2017;4:E349–e356. 28501495 10.1016/S2352-3018(17)30066-8 PMC5555438

[5] Ingle SM May MT Gill MJ . Impact of risk factors for specific causes of death in the first and subsequent years of antiretroviral therapy among HIV-infected patients. Clin Infect Dis. 2014;59:287–297. 24771333 10.1093/cid/ciu261 PMC4073781

[6] Marcus JL Leyden WA Alexeeff SE . Comparison of overall and comorbidity-free life expectancy between insured adults with and without HIV infection, 2000-2016. JAMA Netw Open. 2020;3:e207954. 32539152 10.1001/jamanetworkopen.2020.7954 PMC7296391

[7] Stover J Glaubius R Kassanjee R . Updates to the Spectrum/AIM model for the UNAIDS 2020 HIV estimates. J Int AIDS Soc. 2021;24(suppl 5):e25778. 34546648 10.1002/jia2.25778 PMC8454674

[8] Joint United Nations Programme on HIV/AIDS. Dangerous Inequalities: World AIDS Day Report 2022. Geneva, Switzerland: UNAIDS; 2022. Available at: https://www.unaids.org/sites/default/files/media_asset/dangerous-inequalities_en.pdf. Accessed September 11, 2023.

[9] United Nations, Department of Economic and Social Affairs, Population Division. World Population Prospects 2019: Volume II: Demogaphic Profiles; 2019. Available at: https://population.un.org/wpp/.

[10] Trickey A van Sighem A Stover J . Parameter estimates for trends and patterns of excess mortality among persons on antiretroviral therapy in high-income European settings. AIDS. 2019;33(suppl 3):S271–S281. 31800404 10.1097/QAD.0000000000002387 PMC6919232

[11] Johnson LF Anderegg N Zaniewski E . Global variations in mortality in adults after initiating antiretroviral treatment: an updated analysis of the International epidemiology Databases to Evaluate AIDS cohort collaboration. AIDS. 2019;33(suppl 3):S283–S294. 31800405 10.1097/QAD.0000000000002358 PMC6919233

[12] UNAIDS. AIDSinfo 2023. Available at: https://aidsinfo.unaids.org/. Accessed September 11, 2023.

[13] May MT Ingle SM Costagliola D . Cohort profile: antiretroviral therapy cohort collaboration (ART-CC). Int J Epidemiol. 2014;43:691–702. 23599235 10.1093/ije/dyt010 PMC4052127

[14] Gange SJ Kitahata MM Saag MS . Cohort profile: the North American AIDS Cohort Collaboration on Research and Design (NA-ACCORD). Int J Epidemiol. 2007;36:294–301. 17213214 10.1093/ije/dyl286 PMC2820873

[15] Kowalska JD Friis-Møller N Kirk O . The coding causes of death in HIV (CoDe) project initial results and evaluation of methodology. Epidemiology. 2011;22:516–523. 21522013 10.1097/EDE.0b013e31821b5332

[16] Trickey A May MT Vehreschild J . Cause-specific mortality in HIV-positive patients who survived ten years after starting antiretroviral therapy. PLos One. 2016;11:e0160460. 27525413 10.1371/journal.pone.0160460 PMC4985160

[17] Avenir Health. Spectrum Manual: Spectrum System of Policy Models; 2022. Available at: https://avenirhealth.org/Download/Spectrum/Manuals/SpectrumManualE.pdf. Accessed September 11, 2023.

[18] Projections URGoEMa. UNAIDS Reference Group on Estimates Modelling and Projections. Available at: https://epidem.org/. Accessed September 11, 2023.

[19] Human Mortality Database Max Planck Institute for Demographic Research (Germany), University of California BU, French Institute for Demographic Studies (France). Human Mortality Database 2022. Available at: https://www.mortality.org. Accessed September 11, 2023.

[20] UNAIDS. Spectrum File Request. Available at: https://hivtools.unaids.org/spectrum-file-request/. Accessed September 11, 2023.

[21] Farahani M Mulinder H Farahani A . Prevalence and distribution of non-AIDS causes of death among HIV-infected individuals receiving antiretroviral therapy: a systematic review and meta-analysis. Int J STD AIDS. 2017;28:636–650. 26868158 10.1177/0956462416632428

[22] Fontela C Aguinaga A Moreno-Iribas C . Trends and causes of mortality in a population-based cohort of HIV-infected adults in Spain: comparison with the general population. Sci Rep. 2020;10.8922. 32488053 10.1038/s41598-020-65841-0 PMC7265289

[23] Cheung CC Ding E Sereda P . Reductions in all-cause and cause-specific mortality among HIV-infected individuals receiving antiretroviral therapy in British Columbia, Canada: 2001-2012. HIV Med. 2016;17:694–701. 27279453 10.1111/hiv.12379 PMC5261070

[24] Guaraldi G Orlando G Zona S . Premature age-related comorbidities among HIV-infected persons compared with the general population. Clin Infect Dis. 2011;53:1120–1126. 21998278 10.1093/cid/cir627

[25] De Socio GV Pasqualini M Ricci E . Smoking habits in HIV-infected people compared with the general population in Italy: a cross-sectional study. BMC Public Health. 2020;20.734. 32434482 10.1186/s12889-020-08862-8 PMC7238525

[26] Pettit AC Giganti MJ Ingle SM . Increased non-AIDS mortality among persons with AIDS-defining events after antiretroviral therapy initiation. J Int AIDS Soc. 2018;21, e25031. 29334197 10.1002/jia2.25031 PMC5810321

[27] Deeks SG Tracy R Douek DC. Systemic effects of inflammation on Health during chronic HIV infection. Immunity. 2013;39:633–645. 24138880 10.1016/j.immuni.2013.10.001 PMC4012895

[28] Hsu DC Sereti I. Serious non-AIDS events: therapeutic targets of immune activation and chronic inflammation in HIV infection. Drugs. 2016;76:533–549. 26915027 10.1007/s40265-016-0546-7 PMC5578711

[29] Vourli G Pharris A Cazein F . Are European HIV cohort data within EuroCoord representative of the diagnosed HIV population? AIDS. 2019;33:133–143. 30289806 10.1097/QAD.0000000000002034 PMC6415981

