# Supplementary material for: Estimation of Improvements in Mortality in Spectrum Among Adults With HIV Receiving Antiretroviral Therapy in High-Income Countries
Source: J Acquir Immune Defic Syndr. 2024 Jan 4;95(1 Suppl):e89–96. doi: 10.1097/QAI.0000000000003326 (PMC10769170; doi:10.1097/QAI.0000000000003326)
Supplement: Supplementary file 1 [file qai-95-e89-s001.doc]

Supplementary figure 1: Comparing general population mortality rates per 1000 person-years calculated for Spectrum, 2016-2019, and from mortality.org to match the age, sex, and country distributions of the ART-CC’s population. 

Spectrum general population mortality rates were calculated by applying population-level average mortality rates by sex, age, and country to either the modelled ART population or adjusted to match ART-CC characteristics by age alone or age and country simultaneously.
